# Macroscopic and Microscopic Properties of Cement Paste with Carbon Dioxide Curing

**DOI:** 10.3390/ma15041578

**Published:** 2022-02-20

**Authors:** Jing Zhu, Zijian Qu, Siqi Liang, Baiping Li, Tao Du, Hui Wang

**Affiliations:** 1 College of Civil Engineering and Architecture, Harbin University of Science and Technology, Harbin 150080, China; 1715020417@stu.hrbust.edu.cn (Z.Q.); lsq2313224478@stu.hrbust.edu.cn (S.L.); li2037410612@stu.hrbust.edu.cn (B.L.); 2 School of Mechanics and Civil Engineering, China University of Mining and Technology, Xuzhou 221116, China; dutao@cumt.edu.cn; 3 School of Civil and Environmental Engineering, Ningbo University, Ningbo 315211, China

**Keywords:** carbon dioxide curing, cement-based materials, electrical resistivity, compressive strengths, scanning electron microscope, thermogravimetric analysis

## Abstract

Carbon dioxide is the main component of greenhouse gases, which are responsible for an increase in global temperature. The utilization of carbon dioxide in cement-based materials is an effective way to capture this gas. In this paper, the influence of carbon dioxide curing on the setting time, the electrical resistivity, dry shrinkage ratio, water absorption by unit area and mechanical strengths (flexural and compressive strengths) were determined. The scanning electron microscope, X-ray diffraction and thermogravimetric analysis were obtained to investigate the mechanism of carbonation reaction of cement paste. Water–cement ratios of cement paste were selected to be 0.3, 0.4 and 0.5. Results showed that carbon dioxide curing could accelerate the setting of cement paste. The electrical resistivity decreased with the increasing water–cement ratio and increased with the carbon dioxide curing. Moreover, the evaluation function for the curing age and dry shrinkage rate or the mechanical strengths fit well with the positive correlation quadratic function. The water absorption by unit area increased linearly with the testing time. The carbon dioxide curing led to increasing the mechanical strengths and the dry shrinkage ratio. Meanwhile, the carbon dioxide curing demonstrated a decreasing effect on the water absorption by unit area. The mechanical strengths were improved by the carbon dioxide curing and increased in the form of quadratic function with the curing age. As obtained from the microscopic findings, that the carbon dioxide curing could accelerate the reaction of cement and improve the compactness of cement paste.

## 1. Introduction 

Carbon dioxide is the main culprit of the greenhouse effect. A large amount of carbon dioxide gas will damage the ecological environment. Methods for treating carbon dioxide including plant absorption method, ocean absorption method, agricultural consumption and mechanical capture method. These carbon dioxide treatment methods possessed high cost, low efficiency or poor feasibility [1,2]. In consideration of these reasons, new methods of treating carbon dioxide were needed.

Cement concrete is the most extensive artificial building materials which has been widely used in the construction industry [3,4]. During the production of cement, approximately 5–7% of global anthropogenic carbon dioxide has been emitted by the combustion of fossil fuels and the decomposition of limestone during the production of cement clinker [5,6]. Additionally, other emissions of carbon dioxide induced damage to the ecological environment. As reported in Ravikumar’s research, the utilization of carbon dioxide in cement-based materials is an effective way to capture this gas [7]. The carbon dioxide can react with cement clinker and form calcium carbonate, thus, making the concrete denser [8,9,10]. Prior research has pointed out that proper carbon dioxide curing can improve the mechanical properties and durability of concrete [11,12]. Based on these reasons, carbon dioxide curing on precast concrete materials has been provided for treating the carbon dioxide gas, and simultaneously promoting the properties of cement concrete.

Wang et al. [13] reported that the carbon dioxide curing of a carbonation pressure of 0.5 MPa and carbon dioxide concentration of 95% could be able to increase the compressive strength to 74 MPa at 28th day and 15.8% carbon dioxide was absorbed. Researchers pointed out that the carbon dioxide curing on cement concrete could significantly improve the concrete resistances to sulfate attack, freeze-thaw cycles and chloride penetration. Qin et al. [14] found that the carbon dioxide curing could improve the compressive strength and chloride penetration of cement paste with coal gangue. Moreover, the shrinkage of cement paste with coal gangue was decreased by carbon dioxide curing.

Although carbon dioxide curing can improve the performance of cement concrete, carbon dioxide curing is expensive due to the expensive production of pure CO_2_ and the apparent negative effects of weathering carbonation [15,16]. Researchers confirmed that the carbon dioxide curing with higher concentration could increase the rate of carbonation when the pressure was not above 0.2 MPa [16,17]. Additionally, as reported in some journals, the carbonation rate could be increased by higher water–cement ratios [18,19]. The carbon dioxide curing on the mechanical, performance, the durability and microscopic properties have been provided for several years [20,21,22]. The carbonation of cement-based materials will consume free water inside the cement matrix, eventually degrading the conductivity of the cement-based materials. The electrical parameters have been applied in reflecting the hydration of cement by some scholars [23,24]. However, little attention was paid to the application of electrical properties on the carbonation reaction of cement-based materials. 

This paper aimed to study the influence of carbon dioxide curing on the setting time, the electrical resistivity, dry shrinkage ratio, water absorption by unit area and the mechanical strengths (flexural and compressive strengths). The scanning electron microscope and thermogravimetric analyses were obtained to investigate the mechanism of carbonation reaction of cement paste. The electrical resistivity applied in reflecting the carbonation reaction of cement paste during carbon dioxide curing provided a thought to study the carbonation process of cement paste. This study will offer a way to turn carbon dioxide into resources in the future.

## 2. Experimental

### 2.1. Raw Materials

Ordinary Portland cement with a strength grade of 42.5 MPa was provided by Guangdong Qingxin Cement Co., Ltd., Qingxin, China. The loss on ignition, the fineness, the apparent density and of cement were 5.0%, 1.0% and 3.1 g/cm^3^ respectively. The deionized water was offered by Shanghai Jingchun Water Treatment Technology Co., Ltd., Shanghai, China. The desalination rate of deionized water was 99.99%. The carbon dioxide with the concentration of higher than 99.999% was manufactured by Nanjing Changyuan Industrial Gas Co., Ltd., Nanjing, China. The chemical compositions and particle size distributions of all raw materials are shown in Table 1 and Table 2 respectively.

### 2.2. Specimen Preparation

The specimens were prepared following these steps:

The cement was poured into the water slowly in the NJ-160 cement mixer produced by Shangyu Yueda Instrument Factory, Shaoxing, China and mixed for 2 min with the stirring speed of 140 rpm. Then, the onther stirring speed of 285 rpm and stirring time of 2 min was provided for mixing the fresh samples. After the mixing was finished, the fresh paste was poured into the molds for manufacturing the specimens with sizes of 50 mm × 50 mm × 50 mm and 40 mm × 40 mm × 160 mm. CCB-70B carbonation tank produced by Suzhou Donghua Test Instrument Co., Ltd.,Suzhou, China, was offered for the carbon dioxide curing of cement paste. The concentration of carbon dioxide was 8% by total mass of gas. In this study, the curing environment with 95% relative humidity and 20 °C was the standard curing environment. Table 3 demonstrates the mixing proportion of the specimens in this experiment.

### 2.3. Measurement Methods

#### 2.3.1. The Setting Time Experiment

The testing mold for the setting time of cement paste was circular truncated cone with the top diameter of 60 mm, bottom diameter of 70 mm, and a height of 40 mm. Cement standard consistency setting time tester (standard favicat tester) cement Vicat tester manufactured by Hebei Yiwei Trading Co., Ltd., Cangzhou, China was applied in the determination of the setting time of cement. The experimental process was carried out according to GBT1346-2011 [25].

#### 2.3.2. The Electrical Resistivity Experiment

**Figure 1:**
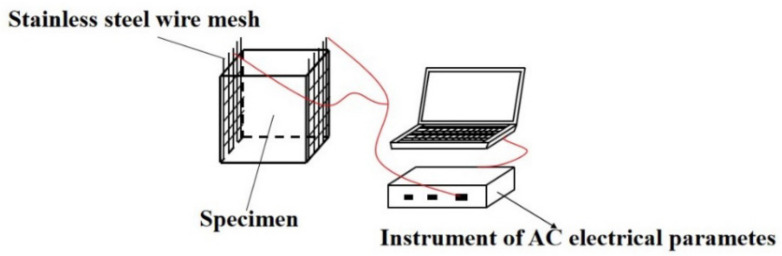
The measurement of AC electrical resistance.

The electrical resistivity was determined by two electrode method. Two pieces of 304 stainless steel mesh with the square hole sizes of 4.75 mm served as two electrode. TH2810D LCR digital electric bridge (Changzhou Tonghui Co., Ltd., Changzhou, China) was provided for the measurement of AC electrical resistivity. The testing frequency of TH2810D LCR digital electric bridge was 10,000 Hz, meanwhile, the testing voltage was 1 V. The electrical resistivity can be calculated by Equation (1):

(1) $$\rho =\frac{RS}{L}$$

where *R* means the electrical resistance of specimen, *S* is the interface area of specimen and *L* is the length of specimen. The measurement of AC electrical resistance was shown in Figure 1. The testing method of the electrical parameters in this section referred to refs. [23,26].

#### 2.3.3. The Experiments of Mechanical Performance and Permeability

**Figure 2:**
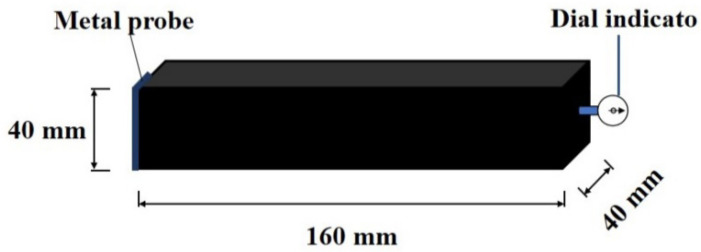
The measurement of dry shrinkage rate.

The YAW-300 microcomputer (Hengruijuan Co., Ltd., Jinan, China) full-automatic universal was used for testing the compressive and flexural strengths of specimens. The loading speeds for compressive and flexural strengths were 2.4 kN/s and 0.05 kN/s respectively. The experiment of mechanical strengths was conducted according to GB/T 17671-1999 Chinese standard [27]. The shrinkage rod of the dial indicator manufactured by Kaiyue Co., Ltd., Cangzhou, China supporting the middle of one end of the rectangular specimen was applied in the determination of dry shrinkage rate. When the length of the specimen changes, the dial indicator reads out the value of the length change. Through this method, the dry shrinkage rate was measured. The measurement of dry shrinkage rate is shown in Figure 2. The measuring environments for the shrinkage rate were standard curing environment (95% relative humidity and 20 °C) and carbon dioxide curing environment (8% by total mass of gas, 65% relative humidity and 20 °C).

Cylinder specimens with size of Φ100 mm × 50 mm were used for the measurement of water absorption by unit area. Before testing, all specimens were desiccated in the vacuum drying oven provided by Shanghai Hecheng Instrument Manufacturing Co., Ltd., Shanghai, China, with a continuous temperature of 60 °C for 4 days. After this process, the lateral sizes of specimens were sealed with epoxy resin to prevent water adsorption. When the epoxy resin was hardened, the bottom surface of each specimen was immersed in water with the depth of 2 cm. The water absorption by unit area can be expressed by Equation (2) as follows:

(2) $$V_{w}={St}^{0.5}+b$$

where *V_w_* (g/m^2^) is the water absorption by unit area, *S* (g/(m^2^/min^0.5^)) is the sorptivity of the material, *t* (min) is the elapsed time and *b* (g/m^2^) is the initial water absorption. The curing condition of all samples is illustrated in Table 4.

#### 2.3.4. Experiments of Thermal Analysis and Scanning Electron Microscopy (SEM)

In order to obtain the thermal analysis curves, these steps can be described as follows:

The samples cured in carbon dioxide curing environment for 1 day, 3 days and 28 days were taken and immersed in the absolute ethanol for 4 days to prevent the hydration of cement. After that, all samples were dried in the vacuum drying oven at the temperature of 60 °C for 4 days before the measurement. The soybean size of hardened cement paste was taken from the inner specimens cured in the carbon dioxide curing environment for 1 day, 3 days and 28 days. The dried samples were sprayed by gold before measurement. Some other samples were filtrated with a 74 µm sieve before thermogravimetric experiment.

The weighed sample powder was placed in an alumina pan of confined space in the thermogravimetric analyzer. The nitrogen atmosphere, whose flow rate is 20 mL/min, was provided as shielding gas. The temperature in the thermogravimetric analyzer ranged from 20 °C to 950 °C. The experimental process of the thermal analysis curves is referred in Refs [24,28]. The soybean size of hardened cement paste sprayed by gold was used for the measurement of scanning electron microscope (SEM). JSM-6360LV scanning electron microscope (Japan electron optics laboratory, Tokyo, Japan) and TGA 4000 thermogravimetric analyzer provided by Perkin Elmer Instrument Co., Ltd., New York, NY, USA were applied in the measurement of thermal analysis curves and SEM photos.

## 3. Results and Discussion

### 3.1. The Setting Time

**Figure 3:**
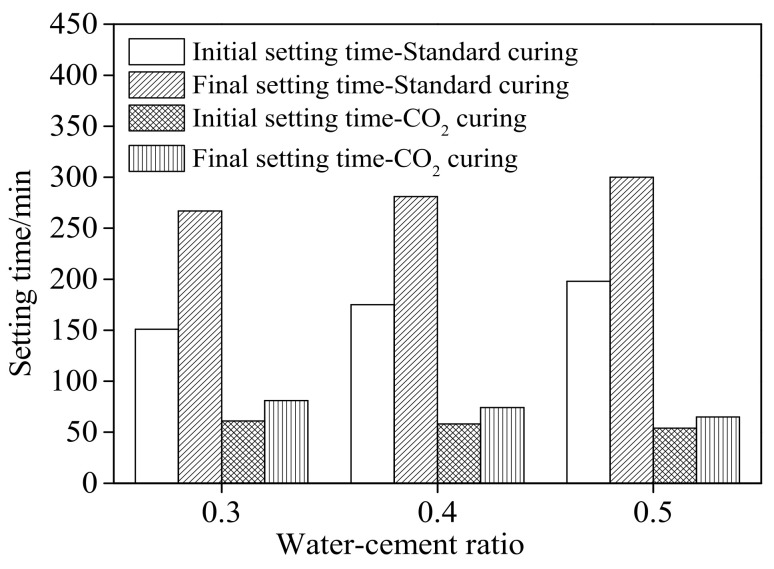
The setting time of cement paste.

**Figure 4:**
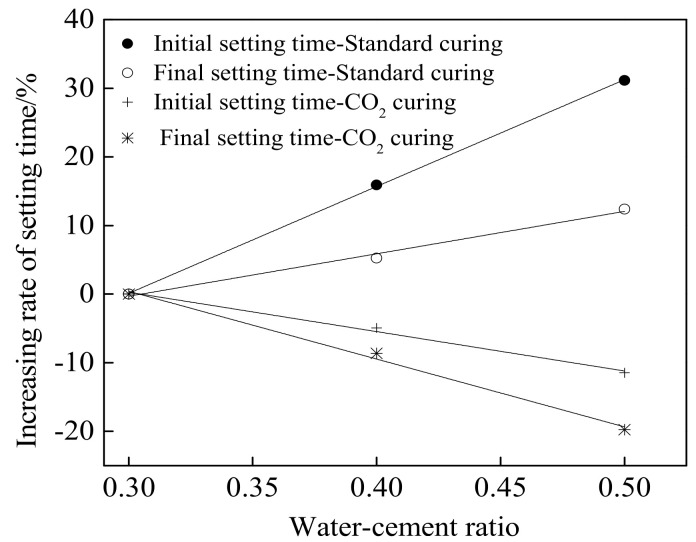
The increasing rate of setting time.

Figure 3 and Figure 4 show the setting time and the following increasing rate of cement paste cured in standard curing environment and CO_2_ curing environment, respectively. Table 5 shows the fitting result of the relationship between the increasing rate of the setting time and the water–cement ratio of cement paste. The setting time includes initial setting (IS) time and final setting (FS) time. As obtained from Figure 3 and Figure 4 and Table 5, the setting time increased with the increasing water–cement ratio and the increasing rate increased linearly when the specimens were cured in a standard curing environment. This was attributed to the fact that cement paste with higher water–cement ratio exhibited more free water leading eventually to delay the setting time [29,30]. However, when the specimens were cured in the CO_2_ curing environment, the setting time decreased, with the decreasing rate corresponding to the linear function with the increasing water–cement ratio due to the fact that the CO_2_ curing accelerate the carbonation reaction leading to decreasing the setting of cement [31,32]. Meanwhile, when CO_2_ curing was provided, the setting time of cement paste with a higher water–cement ratio was lower due to the fact that the carbonation speed of cement paste with a higher water–cement ratio was faster [33,34]. It can be acquired from Table 5, the increasing rate of setting time conformed to the linear function with the water–cement ratio. The fitting degrees were higher than 0.98, which confirmed the accuracy of the fitting function.

### 3.2. The Electrical Resistivity

**Figure 5:**
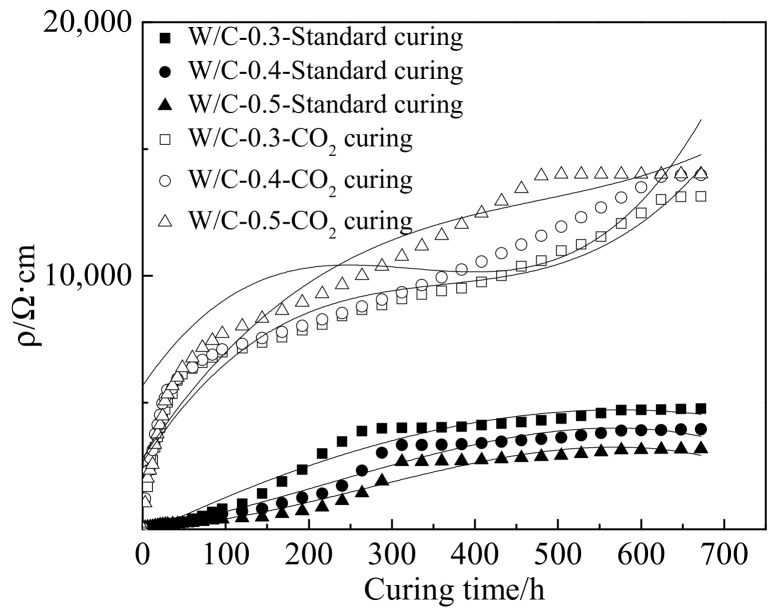
The electrical resistivity of specimens.

Figure 5 shows the electrical resistivity of cement paste cured in standard curing environment and CO_2_ curing environment, respectively. As observed from Figure 5, the electrical resistivity increased in the form of cubic function with the curing time. This was due to the fact that the free water in the pore solution was consumed with the development of cement hydration [35,36]. Therefore, the conductivity was reduced and the electrical resistivity increased with the curing age. Meanwhile, the CO_2_ curing on cement paste could increase the content of calcium carbonate and a dense structure was formed on the hydration products of cement, thus, hindering the migration of conductive ions and increasing the electrical resistivity of cement paste [37,38]. When the specimens were cured in standard curing environment, the electrical resistivity decreased with the increasing water–cement ratio. This was attributed to the fact that free water increased with the increasing water–cement ratio, thus, improving the electrical conduction and decreasing the electrical resistivity [39,40]. However, as shown in Figure 5, the electrical resistivity increased with the increasing water–cement ratio. This could be ascribed to the fact that the rate of carbonation increased with the increasing water–cement ratio, thus, forming more calcium carbonate and preventing the migration of conductive ions leading eventually to increasing the electrical resistivity of cement paste [41,42]. It can be acquired from the result that the CO_2_ curing on cement paste led to decreasing the electrical conduction and increasing the electrical resistivity of cement paste. Table 6 exhibits the values of fitting parameters of all curves. As depicted in Table 6, the fitting degrees of all the curves were higher than 0.94, which proved the accuracy of the fitting equations.

### 3.3. The Results of Mechanical Performance and Permeability

**Figure 6:**
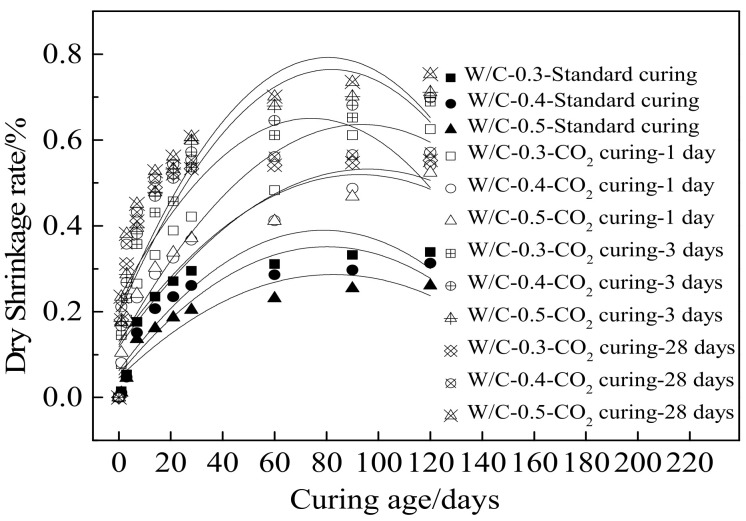
The shrinkage rate of specimens.

In this section, the specimens were cured in CO_2_ for a different amount of time; after curing, they were moved to the standard curing environment for different curing ages. Figure 6 shows the dry shrinkage rate of specimens at different curing ages. As shown in Figure 6, the dry shrinkage ratio of specimens increased rapidly with the curing age ranging from 1 day to 28 days, due to the fact that the free water decreased with the improvement of hydration degree [43,44]. Therefore, the vacancies increased with the improvement of hydration, leading to increasing the dry shrinkage rate. However, when the curing age increased from 28 days to 120 days, the dry shrinkage ratio increased slowly with the increasing curing age. Table 7 shows the fitting results of the relationship between the curing age and dry shrinkage rate. It can be obtained from Table 7, the relationships between the curing age and the dry shrinkage rate satisfied the quadratic function. Moreover, the fitting degrees of all curves were higher than 0.8, signifying correctness of fitting equations. As depicted in Figure 6, higher water–cement ratio led to decreasing the dry shrinkage ratio of specimens when cured in standard curing environment. This was attributed to the fact that the loss rate of free water was decreased by the increasing water–cement ratio thus decreasing the dry shrinkage ratio of specimens [45,46]. Furthermore, the CO_2_ curing could increase the dry shrinkage ratio of specimens, due to the fact that the carbonation will consume free water inside the cement paste, resulting in increasing the shrinkage ratio. When the specimens were cured in CO_2_, a higher water–cement ratio led to the increased shrinkage ratio due to the improvement of carbonation degree [47]. The values of fitting parameters for all fitting curves are displayed in Table 7. The fitting degrees were higher than 0.8, which means that the fitting equations for all curves were reasonable.

**Figure 7:**
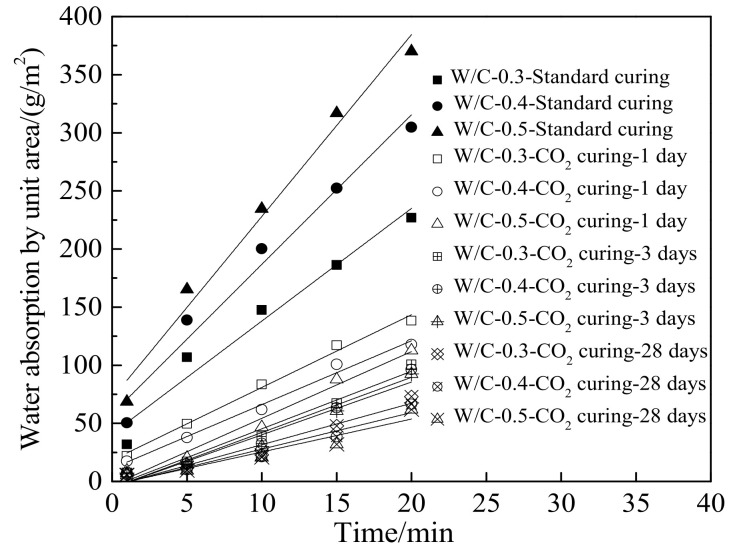
The water absorption by unit area during the testing time.

Figure 7 shows the water absorption by unit area during the testing time. As illustrated in Figure 7, the water absorption by unit area increased linearly with the increasing measuring time. Moreover, the water absorption by unit area was decreased by the decreasing water–cement ratios (cured in standard curing environment) and the increasing CO_2_ curing time. This was attributed to the fact that the decreasing water–cement ratios and the increasing CO_2_ curing time could effectively improve the compactness of the cement paste and reduce the number and volume of pores leading eventually to decreasing the water absorption by unit area [48]. Furthermore, when cured in the CO_2_ curing environment, the influence of the water–cement ratio on the water absorption by unit area was contrary to the standard curing environment due to the enhanced carbonation by the increased water–cement ratio. Table 8 shows the values of fitting parameters for all fitting curves. As demonstrated in Table 8, the fitting degrees are higher than 0.91, which represents the high accuracy of the fitting equation.

**Figure 8:**
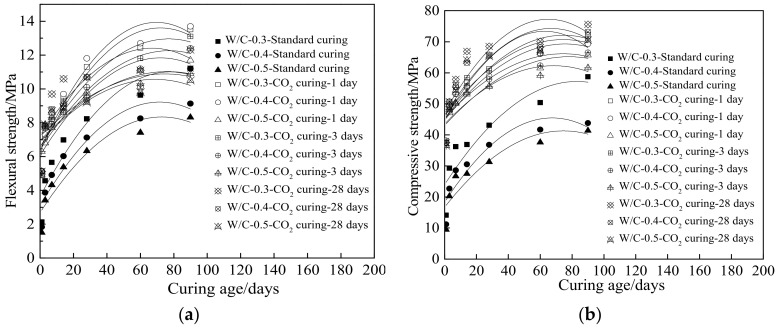
The mechanical strengths of cement paste at different curing age. (**a**) Flexural strength (**b**) Compressive strength.

Figure 8 shows the mechanical strengths (compressive strength and flexural strength) of cement paste. Table 9 shows the fitting results for mechanical strengths. As depicted in Figure 8 and Table 9, the mechanical strengths increased obviously with the curing age, ranging from 1 day to 28 days. Meanwhile, slow increase of mechanical strengths occurred with the curing age, increasing from 28 days to 90 days. It can be observed from Figure 8 that the mechanical strengths were improved by the CO_2_ curing, especially when the curing age was lower than 28 days. When the specimens were cured in the standard curing environment, the mechanical strengths increased with the decreasing water–cement ratio. However, when the CO_2_ curing was provided, the mechanical strengths were improved by CO_2_ curing, and the higher water–cement ratio could enhance the effect of improvement. This was attributed to the fact that higher water–cement ratio led to more pores and loose hydration products, resulting in the decreased mechanical strength [49]. Additionally, the CO_2_ curing could effectively improve the compactness of hydration products, thus, increasing the corresponding mechanical strengths [50]. When the CO_2_ curing was applied, the water–cement ratio was effective for the mechanical strength of cement paste due to the more compact hydration products by CO_2_ curing.

### 3.4. Micro Analysi

**Figure 9:**
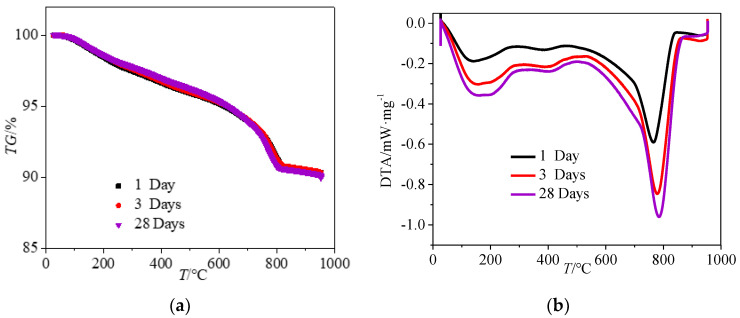
Thermogravimetric analysis curves of specimens with water–cement ratio of 0.3 after CO_2_ curing. (**a**) TG curves of specimens (**b**) DTA curves of specimens.

The thermogravimetric (TG) analysis and differential thermal analysis (DTA) were determined to reflect the hydration products of cement. The testing temperature increased from 20 °C to 950 °C. Figure 9 shows the TG/DTA results of cement pastes with water–cement ratio of 0.3 cured in CO_2_ for 1, 3 and 28 days, respectively. The TG and DTA of all groups can be divided into three steps. The first step was the temperature of 20–150 °C with the mass loss and the thermal entropy losses of the three groups were 0–0.97% and 0–0.37 mW·mg^−1^, respectively, which were caused by the evaporation of free water in cement paste. Moreover, when the temperature ranged from 150 °C to 430 °C continuous mass loss and thermal entropy loss occurred. This was attributed to the fact that the water in the C–S–H pores and the decomposition of C–S–H gel consumed by the hydration and carbonation of cement. Furthermore, when the temperature ranged from 430 °C to 600 °C, the mass loss and the thermal entropy loss decreased rapidly with the increasing temperature. This was attributed to the decomposition of Ca(OH)_2_(CH) crystals. When the temperature was in the region of 600–900 °C, there was a mass loss in the TG curve as well as a significant peak in the DTA curve, which corresponds to the decomposition of calcium carbonate from CO_2_ curing.

**Figure 10:**
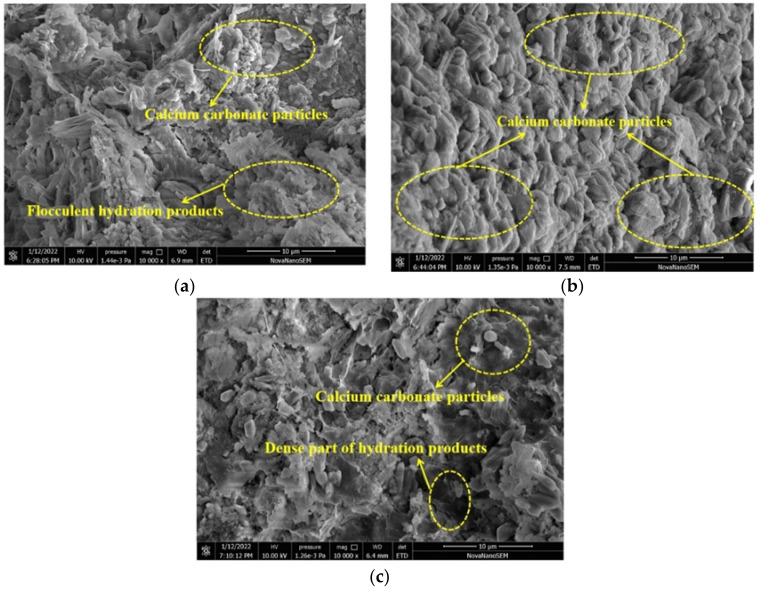
SEM micrographs of specimens cured in carbon dioxide. (**a**) Cured for 1 day (**b**) Cured for 3 days (**c**) Cured for 28 days.

Figure 10 shows the SEM micrographs of specimens cured in CO_2_ for 1, 3, and 28 days, respectively. As shown in Figure 10a, it could be seen that the hydration products at 1 day comprised of needle-like C–S–H gel and flake-like C–H crystals with flocculent phases in between, and the microstructure was mainly a porous and weak cross-linked network. After being cured for 3 days, a denser microstructure and formation of calcium carbonate particles could be observed, as shown in Figure 10b,c. With the increasing CO_2_ curing age, the calcium carbonate particles increased and became compact. This was attributed to the fact that the CO_2_ curing could accelerate the carbonation of cement, leading to the formation of calcium carbonate. The calcium carbonate formed a dense structure inside the cement paste; therefore, the SEM micrographs became denser with the increasing CO_2_ curing age, which agreed well with the strength results discussed in the earlier section.

## 4. Conclusions

In this study, the influences of CO_2_ curing on the hydration and mechanical properties of cement pastes with different water–cement ratios were investigated. The following conclusions can be drawn from the above experimental findings:

The CO_2_ curing led to a decreased setting time of cement paste. The relationship between the setting time and the water–cement ratio can be deduced as a linear function. When cured in standard curing environment, higher water–cement ratio demonstrated a positive effect on the setting time; however, when cured in a CO_2_ curing environment, the effect was the opposite.

The electrical resistivity of cement paste increased with the curing time in a standard curing environment, which followed a cubic function. The carbon dioxide curing facilitated the increase in electrical resistivity by accelerating the carbonation of cement. 

Both flexural and compressive strengths of CO_2_-cured cement pastes were higher than the corresponding standard cured samples; when the water–cement ratio was higher, the enhancing effect was more obvious. The correlations of dry shrinkage rate and mechanical strengths could be expressed through quadratic functions. However, the water absorption by unit area and the testing time conformed to the positive linear function.

TG/DTA analysis and SEM observations concurred that CO_2_ curing process facilitated the formation of calcium carbonate through the consumption of CH which led to a more densified microstructure. Therefore, the improved mechanical properties can be attributed to a higher degree of cement hydration and a more compact microstructure induced by CO_2_ curing. 

## Figures and Tables

**Table 1:** Particle passing percentage of the cementitious materials/%.

	Particle Size/µm	0.3	0.6	1	4	8	64	360
Types								
**P·O Cement**		0	0.33	2.66	15.01	28.77	93.59	100

**Table 2:** Chemical composition of the cementitious materials/%.

Types	SiO_2_	Al_2_O_3_	Fe_2_O_3_	MgO	CaO	SO_3_	T_i2_O
**P·O Cement**	20.86	5.47	3.94	1.73	62.23	2.66	/

**Table 3:** Mixing mass proportion of cement paste.

Cement.	Water	Water Reducing Agent (%)
100	30	0.3
100	40	0.3
100	50	0.3

**Table 4:** The curing condition of the samples.

Types	The Maximum Standard Curing Time/d	The Maximum CO_2_ Curing Time/d
W/C-0.3-Standard curing	28	0
W/C-0.4-Standard curing	28	0
W/C-0.5-Standard curing	28	0
W/C-0.3-CO_2_ curing	0	28
W/C-0.4-CO_2_ curing	0	28
W/C-0.5-CO_2_ curing	0	28
W/C-0.3-CO_2_ curing-1d	89	1
W/C-0.4-CO_2_ curing-1d	89	1
W/C-0.5-CO_2_ curing-1d	89	1
W/C-0.3-CO_2_ curing-3d	87	3
W/C-0.4-CO_2_ curing-3d	87	3
W/C-0.5-CO_2_ curing-3d	87	3
W/C-0.3-CO_2_ curing-28d	62	28
W/C-0.4-CO_2_ curing-28d	62	28
W/C-0.5-CO_2_ curing-28d	62	28

**Table 5:** The fitting results of setting time and w/c.

Equation	Types	*a*	*b*	*R^2^*
t=awc+b	IS-Standard curing	155.629	−46.578	1.000
	FS-Standard curing	61.798	−18.851	0.985
	IS-CO_2_ curing	57.377	17.486	0.986
	FS-CO_2_ curing	30.041	−98.766	0.990

**Table 6:** The fitting results of curing time and w/c.

Equation	Types	*a*	*b*	*c*	*d*	*R^2^*
$\rho =at^{3}+bt^{2}+ct+d$	W/C-0.3-Standard curing	−4.26 × 10^−6^	−0.010	15.771	−212.747	0.978
	W/C-0.4-Standard curing	−2.68 × 10^−5^	0.018	5.421	2.578	0.979
	W/C-0.5-Standard curing	−3.11 × 10^−5^	0.026	1.062	85.999	0.974
	W/C-0.3-CO_2_ curing	1.19 × 10^−4^	−0.130	50.729	2641.442	0.941
	W/C-0.4-CO_2_ curing	1.62 × 10^−4^	−0.158	48.327	5660.494	0.975
	W/C-0.5-CO_2_ curing	6.33 × 10^−5^	−0.091	50.154	2807.444	0.950

**Table 7:** The fitting results of dry shrinkage rate and the curing age.

Equation	Types	*a*	*b*	*c*	*R^2^*
$\frac{\Delta L}{L}=at^{2}+bt+c$	W/C-0.3-Standard curing	−5.26 × 10^−5^	0.008	0.0624	0.871
	W/C-0.4-Standard curing	−4.63 × 10^−5^	0.007	0.053	0.895
	W/C-0.5-Standard curing	−3.53 × 10^−5^	0.006	0.046	0.910
	W/C-0.3-CO_2_ curing-1d	−5.87 × 10^−5^	0.011	0.123	0.864
	W/C-0.4-CO_2_ curing-1d	−4.50 × 10^−5^	0.009	0.118	0.837
	W/C-0.5-CO_2_ curing-1d	−4.53 × 10^−5^	0.008	0.131	0.898
	W/C-0.3-CO_2_ curing-3d	−8.46 × 10^−5^	0.014	0.195	0.888
	W/C-0.4-CO_2_ curing-3d	−9.04 × 10^−5^	0.015	0.203	0.891
	W/C-0.5-CO_2_ curing-3d	−7.67 × 10^−5^	0.011	0.229	0.848
	W/C-0.3-CO_2_ curing-28d	−1.03 × 10^−4^	0.003	0.152	0.856
	W/C-0.4-CO_2_ curing-28d	−1.11 × 10^−4^	0.0012	0.141	0.871
	W/C-0.5-CO_2_ curing-28d	−1.02 × 10^−4^	0.0015	0.127	0.864

**Table 8:** The fitting results of water absorption by unit area and the curing age.

Equation	Types	*a*	*b*	*R^2^*
$V_{W}=at+b$	W/C-0.3-Standard curing	9.697	40.981	0.952
	W/C-0.4-Standard curing	12.875	57.984	0.966
	W/C-0.5-Standard curing	15.648	71.453	0.978
	W/C-0.3-CO_2_ curing-1d	6.257	18.288	0.990
	W/C-0.4-CO_2_ curing-1d	5.491	11.159	0.985
	W/C-0.5-CO_2_ curing-1d	5.744	−3.151	0.973
	W/C-0.3-CO_2_ curing-3d	4.954	−4.271	0.967
	W/C-0.4-CO_2_ curing-3d	4.752	−5.422	0.948
	W/C-0.5-CO_2_ curing-3d	4.546	−5.358	0.941
	W/C-0.3-CO_2_ curing-28d	3.568	−4.057	0.931
	W/C-0.4-CO_2_ curing-28d	3.641	3.145	0.911
	W/C-0.5-CO_2_ curing-28d	3.0108	2.836	0.920

**Table 9:** The fitting results for mechanical strengths.

Equation	Types	*a*	*b*	*c*	*R^2^*
$f_{t}=at^{2}+bt+c$	W/C-0.3-Standard curing	−0.00122	0.190	3.632	0.864
	W/C-0.4-Standard curing	−0.0012	0.172	3.085	0.889
	W/C-0.5-Standard curing	−0.00107	0.156	2.677	0.858
	W/C-0.3-CO_2_ curing-1d	−0.00141	0.203	6.323	0.881
	W/C-0.4-CO_2_ curing-1d	−0.00155	0.216	6.403	0.903
	W/C-0.5-CO_2_ curing-1d	−0.00112	0.161	6.065	0.874
	W/C-0.3-CO_2_ curing-3d	−0.00108	0.166	6.567	0.880
	W/C-0.4-CO_2_ curing-3d	−9.79 × 10^−4^	0.151	6.428	0.889
	W/C-0.5-CO_2_ curing-3d	−6.67 × 10^−4^	0.0993	7.191	0.860
	W/C-0.3-CO_2_ curing-28d	−0.00115	0.154	7.238	0.833
	W/C-0.4-CO_2_ curing-28d	−7.04 × 10^−4^	0.107	6.931	0.815
	W/C-0.5-CO_2_ curing-28d	−7.89 × 10^−4^	0.108	6.854	0.842
	W/C-0.3-Standard curing	−0.00482	0.80118	23.927	0.901
$f_{cu}=at^{2}+bt+c$	W/C-0.4-Standard curing	−0.00598	0.807	18.239	0.854
	W/C-0.5-Standard curing	−0.00443	0.661	16.657	0.849
	W/C-0.3-CO_2_ curing-1d	−0.00488	0.729	44.868	0.848
	W/C-0.4-CO_2_ curing-1d	−0.00432	0.652	44.664	0.882
	W/C-0.5-CO_2_ curing-1d	−0.00365	0.569	42.973	0.873
	W/C-0.3-CO_2_ curing-3d	−0.00442	0.658	46.221	0.898
	W/C-0.4-CO_2_ curing-3d	−0.00374	0.557	45.419	0.842
	W/C-0.5-CO_2_ curing-3d	−0.00402	0.550	43.444	0.805
	W/C-0.3-CO_2_ curing-28d	−0.0071	0.925	47.134	0.828
	W/C-0.4-CO_2_ curing-28d	−0.00629	0.841	46.150	0.825
	W/C-0.5-CO_2_ curing-28d	−0.00724	0.910	44.771	0.867

## Data Availability

Not applicable.
